# Male sex is a risk factor for detrusor pressure jeopardising the upper urinary tract in patients with spinal cord injury

**DOI:** 10.1111/bju.16925

**Published:** 2025-09-18

**Authors:** Veronika Birkhäuser, Collene E. Anderson, Marko Kozomara, Martin W.G. Brinkhof, Oliver Gross, Lorenz Leitner, Martina D. Liechti, Ulrich Mehnert, Lara Stächele, Thomas M. Kessler

**Affiliations:** ^1^ Department of Neuro‐Urology, Balgrist University Hospital University of Zürich Zürich Switzerland; ^2^ Swiss Paraplegic Research Nottwil Switzerland; ^3^ Faculty of Health Sciences and Medicine University of Lucerne Lucerne Switzerland; ^4^ Department of Urology Cantonal Hospital Lucerne Lucerne Switzerland

**Keywords:** urodynamics, spinal cord injuries, urinary bladder, neurogenic, urinary bladder, overactive, sex characteristics

## Abstract

**Objective:**

To evaluate sex differences in maximum storage detrusor pressure (Pdet_max_ storage), focusing on Pdet_max_ storage ≥40 cmH_2_O, an established risk factor for upper urinary tract damage, within the first year after spinal cord injury (SCI).

**Patients and Methods:**

A cohort of patients with neurogenic lower urinary tract dysfunction due to acute traumatic or ischaemic SCI, managed according to the European Association of Urology Guidelines on Neuro‐Urology, prospectively underwent urodynamic investigations at 1, 3, 6 and 12 months after SCI at a university SCI centre. Pearson's chi‐squared tests and multivariable regression analyses were used to compare outcomes between females and males.

**Results:**

Of 97 patients, 34% were female. Within the first year after SCI, 9% of females presented with a Pdet_max_ storage ≥40 cmH_2_O, compared to 55% of males (*P* < 0.001). Females had lower Pdet_max_ storage than males, with a grand mean (standard deviation [SD]) of 13 (9) vs 30 (20) cmH_2_O (coefficient = 18 cmH_2_O, 95% confidence interval [CI] 13–24 cmH_2_O; *P* < 0.001). Females also had lower detrusor overactivity leak‐point pressure (DOLPP) than males, with a grand mean (SD) of 15 (4) vs 35 (21) cmH_2_O (coefficient = 20 cmH_2_O, 95% CI 11–29 cmH_2_O; *P* < 0.001). The cumulative incidence of antimuscarinic therapy and reported urinary incontinence was similar between sexes (female vs male: 55% vs 70%, *P* = 0.12; 53% vs 43%, *P* = 0.42, respectively).

**Conclusions:**

Females rarely reached a Pdet_max_ storage ≥40 cmH_2_O during the first year after SCI and had lower Pdet_max_ storage and DOLPP than males. However, the prevalence of urinary incontinence was similar between the sexes, suggesting the need for a more sex‐tailored approach to patient management.

**Abbreviations**
AIS: American Spinal Injury Association Impairment Scale
ALPP: abdominal leak‐point pressure
DO(I): detrusor overactivity (incontinence)
DOLPP: DO leak‐point pressure
EMSCI: European Multicenter Study about Spinal Cord Injury
Q1‐Q3: first and third quartiles
LUT: lower urinary tract
NLUTD: neurogenic LUT dysfunction
(a)OR: (adjusted) odds ratio
Pdet: detrusor pressure
Pdet_max_
: maximum Pdet
SCI: spinal cord injury
UDI: urodynamic investigation
(S)UI: (stress) urinary incontinence
UUT(D): upper urinary tract (deterioration)

## Introduction

Spinal cord injuries (SCI) frequently affect the nervous system controlling the lower urinary tract (LUT) resulting in neurogenic LUT dysfunction (NLUTD). Alongside various symptoms, such as urinary incontinence (UI), which negatively impact the health‐related quality of life, the most severe long‐term sequelae of NLUTD is renal deterioration [1, 2]. A high maximum storage detrusor pressure (Pdet_max_ storage) with or without VUR is considered the most important risk factor for renal failure [3, 4, 5]. However, there is no universally accepted definition for high Pdet_max_ storage in individuals affected with SCI. In clinical practice, the most widely used cut‐off is ≥40 cmH_2_O [6, 7]. Nevertheless, there are heterogeneous findings on a potential harmful Pdet threshold in the SCI population. A retrospective study identified a Pdet_max_ storage ≥75 cmH_2_O as an independent risk factor for upper urinary tract deterioration (UUTD) in patients with SCI [8]. In contrast, another study reported a significant association between a Pdet_max_ storage ≥15 cmH_2_O and UUTD [9]. These studies share the limitation of not investigating potential differences in thresholds for Pdet_max_ storage between females and males. Findings from the SCI population indicate that male sex may pose an increased risk of UUT complications and renal failure [5]. Additionally, male sex has been identified as a risk factor for the occurrence of Pdet_max_ storage ≥40 cmH_2_O within the first year after SCI [10]. However, no studies have assessed how Pdet_max_ storage varies between females and males with SCI. Therefore, the objective of this study was to evaluate the relationship between sex and Pdet_max_ storage, UI symptoms, and antimuscarinic use during the first year after SCI using urodynamic data from the prospective, longitudinal, population‐based European Multicenter Study about Spinal Cord Injury (EMSCI).

## Patients and Methods

We followed the Strengthening the Reporting of Observational Studies in Epidemiology (STROBE) guidelines to enhance the quality of reporting [11].

### Patients

The study involved patients from the prospective, longitudinal, population‐based EMSCI (www.emsci.org) who underwent urodynamic investigations (UDIs) at the Balgrist University Hospital, Zürich, a specialised SCI rehabilitation centre. Eligible participants were adults (aged ≥18 years) with a single‐event traumatic or ischaemic SCI who could undergo EMSCI neurological assessments within 6 weeks of SCI. Patients with severe brain injury, cognitive impairment, pre‐existing dementia, polyneuropathy, or peripheral nerve lesion above the level of SCI were excluded. Data were collected from January 2014 to December 2019, with a 77% EMSCI participation rate. The EMSCI assessments were performed at four standardised time points: 1 month (Days 16–40), 3 months (Days 70–98), 6 months (Days 150–186), and 12 months (Days 300–546) after SCI. If changes or new symptoms, such as UI, occurred, an additional UDI was performed as indicated. The study followed the Declaration of Helsinki, with written informed consent, and was approved by the Zürich Cantonal Ethics Committee (PB_2016–00293, EK‐03/2004).

### Neuro‐Urological Assessment and Management

Neuro‐urological assessments were conducted based on the recommendations by Panicker et al. [2]. Video‐UDIs followed a standardised UDI protocol outlined in a previous publication [12] and adhered to the ICS recommendations on good urodynamic practices [13]. All definitions, methods, and units conformed to ICS standards [14]. To minimise assessor bias, UDIs were randomly assigned to two experienced neuro‐urologists for evaluation. Neuro‐urological management followed the European Association of Urology (EAU) Guidelines on Neuro‐Urology, with antimuscarinic medication used as first‐line treatment for unfavourable UDI findings, particularly high Pdet_max_ storage and/or storage symptoms. Intradetrusor onabotulinumtoxinA injections were utilised as a second‐line option [1]. For patients unable to achieve spontaneous voiding, voiding dysfunction was preferably managed by intermittent self‐catheterisation. If that was not feasible, suprapubic catheterisation was utilised. Antibiotic prophylaxis was not administered prior to the UDI in patients with asymptomatic bacteriuria.

### Study Measures

Outcome measures included UDI findings from filling cystometry (detrusor overactivity [DO]; DO leak‐point pressure [DOLPP], sitting position only; DO incontinence [DOI]; stress UI [SUI]; abdominal leak‐point pressure [ALPP]; and Pdet_max_ storage). Information on bladder emptying method, medication usage, UI symptoms, and renal insufficiency was extracted from the medical history.

Patient information (sex, age) and SCI characteristics (e.g., aetiology) were obtained from clinical records by EMSCI. Neurological function, including neurological level and SCI severity (using the American Spinal Injury Association [ASIA] Impairment Scale [AIS] Grade), was evaluated according to the International Standards for Neurological Classification of Spinal Cord Injury (ISNCSCI) at all EMSCI time points [15].

### Statistical Analyses

Statistical analyses were performed using Stata, version 18.0 (StataCorp, College Station, TX, USA). Continuous variables are generally displayed as medians and quartiles (Q1‐Q3, first and third quartiles), grand means (SDs) are used to display Pdet over all time points. Uni‐ and multivariable logistic regression analyses were used to examine the relationship between sex and Pdet_max_ storage ≥40 cmH_2_O, UI symptoms reported in the medical history, and antimuscarinic therapy during the first year after SCI. The relationships between sex, Pdet_max_ storage, and DOLPP (specified as continuous variables) were investigated using uni‐ and multivariable linear mixed‐effects regression analyses stratified on antimuscarinic status at the time of UDI and excluding 26 UDI sessions where the participant had intradetrusor onabotulinumtoxinA injections at <9 months before the UDI. For mixed‐effects analyses, the data were structured in long format, with each row corresponding to a single filling. As both Pdet_max_ storage and DOLPP were heavily right‐skewed, a natural log (ln) transformation was applied, and the results were displayed as marginal predictions that were reverse‐transformed to the original linear scale.

The strategy for multivariable model building was informed by a causal diagram (Fig. S1). All adjusted models included age at SCI, categorised neurological level, and SCI severity (AIS Grade), with mixed‐effects analyses also adjusted for time to UDI (in months) and filling (1 or 2). Additionally, the adjusted model for Pdet_max_ storage included information on DOI and SUI during the filling (yes/no), and position during UDI (sitting/lying down). Interactions between sex and all covariates were tested, as all covariates were assumed to be mediators on the causal pathway between sex and the respective outcome, as were interactions between neurological level and AIS Grade. Sex‐stratified analyses were used to further investigate risk factors; however, the female group was generally restricted to basic descriptive analyses owing to powering issues. Linearity of continuous predictor variables was assessed. In five patients where the 1‐month neurological assessment was missing or not testable, the next available observation was used to impute information. For logistic regression analyses examining the status during the entire first year after SCI, multiple imputation using chained equations [16] was used to impute the outcome data for persons who did not return for the 1‐year follow‐up (*n* = 24) and who had not shown or reported the outcome of interest at the time of loss to follow‐up. Loss to follow‐up in this cohort has previously been described in detail [17].

## Results

The study population consisted of 97 patients, of whom 33 (34%) were female. Patient characteristics stratified by sex are summarised in Table 1. Females were generally older, more likely to have UI symptoms at the time of SCI and had less severe injuries than males. The patients underwent a total of 342 UDIs (females *n* = 113, males *n* = 229). UDI filling cystometry findings, LUT management, UI symptoms at the time of UDI, and evidence of renal insufficiency at the time of UDI stratified by sex are displayed in Table 2. Full UDI findings can be found in the supplement (Table S1).

**Table 1:** Characteristics of the study population at baseline, stratified by sex.

Characteristic [% missing]	Female, *N* = 33	Male, *N* = 64	*P*
*Continuous variable, median (* *Q1‐Q3* *)*			
**Age at SCI, years**	63 (51–74)	56 (36–65)	0.043
**Duration of rehabilitation stay, days**	99 (51–143)	123 (61–178)	0.22
*Categorical variable, n (%)*			
**UI prior to SCI**			
Yes	8 (24)	0 (0)	<0.001
No	21 (64)	54 (84)	
Unknown	4 (12)	10 (16)	
**Age (years) at SCI**			
18–30	1 (3)	9 (14)	0.026
31–45	7 (21)	11 (17)	
46–60	7 (21)	21 (33)	
61–75	11 (33)	21 (33)	
≥76	7 (21)	2 (3)	
**SCI aetiology**			
Traumatic SCI	20 (61)	55 (86)	<0.01
Ischaemic SCI	13 (39)	9 (14)	
**Neurological level [5** * **]**			
Cervical (C1–C8)	12 (36)	31 (48)	0.34
Thoracic (T1–T12)	16 (48)	21 (33)	
Lumbar (L1–L5)	5 (15)	12 (19)	
**AIS Grade [5** * **]**			
A	3 (9)	18 (28)	0.022
B	1 (3)	9 (14)	
C	6 (18)	10 (16)	
D	23 (70)	27 (42)	
**Duration of rehabilitation stay (days)**			
1–60	11 (33)	16 (25)	0.75
61–120	9 (27)	15 (23)	
121–180	7 (21)	17 (27)	
≥181	6 (18)	16 (25)	

* In five patients missing the neurological assessment at baseline (~1 month after SCI), level and AIS Grade are based on data from the next available time point.

**Table 2:** UDI filling cystometry findings, LUT management, and LUTS at the time of UDI, stratified by sex.

Parameter	1‐month follow‐up		3‐month follow‐up		6‐month follow‐up		12‐month follow‐up	
	Female	Male	Female	Male	Female	Male	Female	Male
**UDI performed,** ***n*** **(%)**	31 (94)	59 (92)	30 (91)	55 (86)	26 (79)	49 (77)	23 (70)	50 (78)
**Time from SCI to UDI, days, median (** **Q1‐Q3** **)**	30 (24–36)	30 (24–37)	83 (78–89)	86 (80–91)	174 (169–177)	175 (170–177)	364 (357–381)	366 (355–377)
**Sitting position during UDI,** ***n*** **(%)**	24 (77)	33 (56)	23 (77)	38 (69)	23 (88)	37 (76)	21 (91)	37 (74)
**DO present**, ***n*** **(%)**	19 (61)	45 (76)	15 (50)	48 (87)	15 (58)	46 (94)	15 (65)	42 (84)
DO start volume, mL, median (Q1‐Q3)	210 (155–490)	260 (205–385)	205 (200–375)	363 (200–540)	305 (135–415)	373 (185–470)	395 (185–460)	380 (185–495)
**DOI present,** ***n*** **(%)**	8 (26)	24 (41)	8 (27)	15 (27)	8 (31)	21 (43)	4 (17)	12 (24)
DOLPP, cmH_2_O, median (Q1‐Q3)	17 (13–19)	35 (25–69)	21 (12–22)	33 (17–49)	14 (13–15)	33 (19–46)	11 (4–12)	27 (13–37)
Volume leaked (DOI), mL, median (Q1‐Q3)	85 (20–400)	50 (20–89)	75 (38–229)	40 (33–60)	75 (43–98)	24 (10–48)	108 (25–303)	45 (13–56)
**SUI present,** ***n*** **(%)**	4 (13)	2 (3)	4 (13)	1 (2)	2 (8)	3 (6)	3 (13)	3 (6)
ALPP, cmH_2_O, median (Q1‐Q3)	23 (2–81)	70 (70–70)	5 (1–10)	0 (0–0)	19 (1–33)	32 (7–57)	12 (11–16)	10 (6–59)
Volume leaked (SUI), mL, median (Q1‐Q3)	45 (20–48)	5 (5–5)	20 (13–60)	15 (15–15)	40 (5–45)	28 (20–70)	29 (15–45)	21 (9–30)
**Pdet**_**max**_ **storage, cmH**_**2**_ **O, median** **(** **Q1‐Q3** **)**	9 (4–13)	28 (9–42)	10 (4–17)	28 (14–45)	12 (4–21)	31 (16–39)	15 (4–18)	25 (15–37)
**Pdet**_**max**_ **≥40 cmH**_**2**_ **O,** ***n*** **(%)**	1 (3)	20 (34)	1 (3)	17 (31)	2 (8)	22 (45)	0 (0)	11 (22)
**Medications for the LUT,** ***n*** **(%)**								
Antimuscarinics	0 (0)	0 (0)	7 (23)	21 (38)	9 (35)	24 (49)	6 (26)	25 (50)
Alpha‐blockers	0 (0)	4 (7)	4 (13)	10 (18)	4 (15)	8 (16)	3 (13)	10 (20)
Intradetrusor onabotulinumtoxinA injections*	0 (0)	0 (0)	0 (0)	0 (0)	1* (4)	5* (10)	3* (13)	9* (18)
**Medications potentially influencing the LUT,** ***n*** **(%)**								
Antidepressants/neuroleptics (+ other)	10 (32)	23 (39)	12 (40)	25 (45)	11 (42)	27 (55)	10 (43)	28 (56)
Opioids	11 (35)	26 (44)	11 (37)	25 (45)	9 (35)	20 (41)	9 (39)	19 (38)
**Bladder emptying method,** ***n*** **(%)**								
Indwelling catheter	21 (68)	45 (76)	12 (40)	23 (42)	7 (27)	21 (43)	5 (22)	19 (38)
Transurethral	21 (68)	39 (66)	7 (23)	4 (7)	0 (0)	1 (2)	0 (0)	0 (0)
Suprapubic	0 (0)	6 (10)	5 (17)	19 (35)	7 (27)	20 (41)	5 (22)	19 (38)
Intermittent self‐catheterisation	2 (6)	1 (2)	3 (10)	10 (18)	3 (12)	8 (16)	3 (13)	11 (22)
Combined (spontaneous voiding + catheter)	3 (10)	1 (2)	5 (17)	9 (16)	6 (23)	10 (20)	4 (17)	8 (16)
Spontaneous voiding	5 (16)	12 (20)	10 (33)	13 (24)	10 (38)	10 (20)	11 (48)	12 (24)
**UI symptoms (medical history),** ***n*** **(%)**								
Yes	10 (32)	4 (7)	9 (30)	9 (16)	9 (35)	14 (29)	6 (26)	8 (16)
No	19 (61)	45 (76)	20 (67)	44 (80)	16 (62)	31 (63)	15 (65)	37 (74)
Unknown	2 (6)	10 (17)	1 (3)	2 (4)	1 (4)	4 (8)	2 (9)	5 (10)
**Renal insufficiency,** ***n*** **(%)**								
Yes, chronic	3 (10)	2 (3)	4 (13)	2 (4)	2 (8)	2 (4)	1 (4)	2 (4)
Evidence of insufficiency, possibly transient^†^	5 (16)	7 (12)	4 (13)	9 (16)	4 (15)	4 (8)	2 (9)	4 (8)
No	23 (74)	49 (83)	18 (60)	38 (69)	6 (23)	30 (61)	4 (17)	24 (48)
Unknown	0 (0)	1 (2)	4 (13)	6 (11)	14 (54)	13 (27)	16 (70)	20 (40)

* Cumulative number of persons who received intradetrusor onabotulinumtoxinA injections at this time point.

^†^ Evidence based on creatinine and/or cystatin C levels from blood tests.

Within the first year after SCI, a Pdet_max_ storage ≥40 cmH_2_O was observed in 9% (three of 33) of females in contrast to 55% (35 of 64) of males (*P* < 0.001) (stratified by time point in Table 2). In uni‐ and multivariable regression analyses males were at a higher risk of a Pdet_max_ storage ≥40 cmH_2_O compared to females with an adjusted odds ratio (aOR) of 12.01 (95% CI 3.04–47.02; *P* < 0.001; Table 3). The three females with Pdet_max_ storage ≥40 cmH_2_O were aged 45–76 years. Their SCI characteristics were as follows: C6 AIS Grade B, T8 AIS Grade D (both no known UI before SCI and no SUI during the first year after SCI), and T9 AIS Grade D (with UI before SCI and SUI).

**Table 3:** Factors associated with Pdet_max_ ≥40 cmH_2_O during the storage phase, UI symptoms, and antimuscarinics during the first year after SCI (multiple imputation analysis).

Determinant	Pdet_max_ ≥40 cmH_2_O, OR (95% CI)	*P*	Pdet_max_ ≥40 cmH_2_O, aOR (95% CI)	*P*	UI symptoms, OR (95% CI)	*P*	UI symptoms, aOR (95% CI)	*P*	Antimuscarinics, OR (95% CI)	*P*	Antimuscarinics, aOR (95% CI)	*P*
**Sex**												
Female	REF	<0.01	REF	<0.001	REF	0.26	REF	0.52	REF	0.022	REF	0.11
Male	8.62 (2.33–31.90)		12.01 (3.04–47.42)		0.59 (0.23–1.47)		0.69 (0.23–2.11)		3.24 (1.18–8.92)		2.64 (0.80–8.65)	
**Age at SCI**	1.00 (0.97–1.03)	0.91	1.01 (0.98–1.05)	0.43	1.02 (0.99–1.05)	0.15	1.02 (0.99–1.05)	0.17	1.01 (0.99–1.04)	0.32	1.02 (0.99–1.05)	0.23
**Neurological level** *												
Cervical	2.41 (0.61–9.51)	0.20	4.60 (0.77–27.43)	0.21	0.92 (0.27–3.06)	0.09	0.48 (0.11–2.09)	0.20	1.21 (0.31–4.70)	0.96	1.40 (0.23–8.57)	0.93
Thoracic	1.05 (0.27–4.09)		3.08 (0.51–18.60)		2.69 (0.75–9.67)		1.26 (0.28–5.62)		1.15 (0.28–4.69)		1.29 (0.21–8.05)	
Lumbar	REF		REF		REF		REF		REF		REF	
**AIS Grade** *												
A	1.01 (0.32–3.13)	0.57	0.48 (0.13–1.77)	0.26	2.36 (0.73–7.61)	0.051	2.30 (0.58–9.05)	0.11	11.69 (1.41–96.90)	0.039	8.57 (1.04–70.76)	0.10
B/C	1.78 (0.58–5.47)		2.02 (0.44–9.38)		0.42 (0.13–1.33)		0.37 (0.09–1.52)		2.49 (0.71–8.69)		2.32 (0.48–11.21)	
D	REF		REF		REF		REF		REF		REF	

Results from uni‐ and multivariable logistic regression analyses with presence of the respective outcome coded as ‘1’. Age at injury, neurological level, and AIS Grade are all potential mediators for the effect of sex on the respective outcomes.

* Neurological level and AIS Grade reflect neurological status within 40 days of SCI. In five patients with missing data at the 1‐month time point, information was taken from the next time point with available data.

**Fig. 1:**
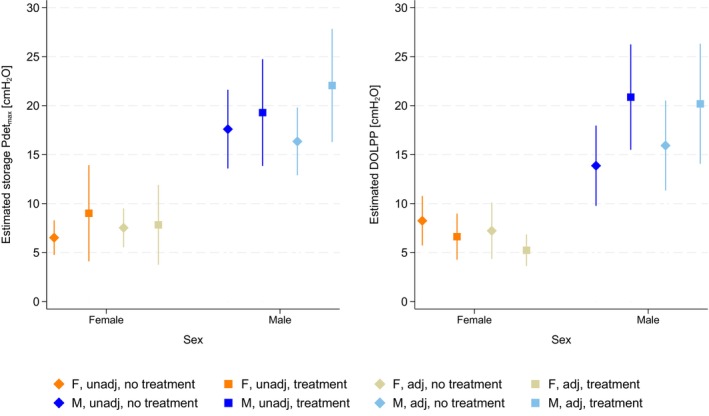
Estimated Pdet_max_ during the storage phase and DOLPP for females and males. Marginal predictions from uni‐ and multivariable linear mixed‐effects regression analyses stratified on use of antimuscarinic medication on the date of UDI, excluding measurements where the participant had intradetrusor onabotulinumtoxinA injections in the 9 months before UDI. Storage Pdet_max_ no antimuscarinic treatment group: *n* = 97 patients, with 439 urodynamic fillings; storage Pdet_max_ antimuscarinic treatment group: *n* = 50 patients and 174 urodynamic fillings. DOLPP no antimuscarinic treatment group: *n* = 29 patients with 60 urodynamic fillings, DOLPP antimuscarinic treatment group: *n* = 16 patients with 39 urodynamic fillings. adj, adjusted; F, female; M, male; unadj, unadjusted.

Females presented with lower Pdet_max_ storage than males on average over all time points, with a grand mean (SD) of 13 (9) vs 30 (20) cmH_2_O (coefficient = 18 cmH_2_O, 95% CI 13–24 cmH_2_O; *P* < 0.001). In uni‐ and multivariable regression analyses stratified on antimuscarinic use at the time of UDI, with Pdet_max_ storage specified as a continuous variable, on average females had a lower Pdet_max_ storage than males irrespective of antimuscarinic use and adjustment for other covariates (Fig. 1, Table S2, all *P* < 0.05). Similarly, females had a lower DOLPP than males, with a grand mean (SD) of 15 (4) vs 35 (21) cmH_2_O (coefficient = 20 cmH_2_O, 95% CI 11–29 cmH_2_O; *P* < 0.001; Fig. 1, Table S3, all *P* < 0.05). However, there was no statistically significant difference in the cumulative incidence of DOI within the first year between the sexes (female vs male: 48% (16 of 33) vs 59% (38 of 64), *P* = 0.31). Regression analyses further supported this finding (males vs females aOR 1.97, 95% CI 0.67–5.81; *P* = 0.22; Table S4).

The prevalence of SUI during UDI was higher in females compared to males, at 21% (seven of 33, three with UI symptoms prior to SCI) vs 6% (four of 64) (*P* = 0.028). On average over all time points the ALPPs were lower in females than males, with a grand mean (SD) of 15 (5) vs 35 (21) cmH_2_O; however, the difference was not statistically significant (*P* = 0.54). Only one of 42 fillings (2%) with SUI present also reached a Pdet_max_ storage ≥40 cmH_2_O.

The cumulative incidence of UI symptoms was similar between sexes, reported by 53% (17 of 33) of females and 43% (27 of 63) of males (*P* = 0.42). See Table 2 for reported symptoms stratified by time point, and Table 3 for regression results. Six of the eight females with UI symptoms prior to SCI had UI symptoms during the first year after SCI. Antimuscarinic therapy was used by 55% (18 of 33) of females and 70% (45 of 64) of males (*P* = 0.12; Table 3). Univariable regression analysis pointed towards a total effect of sex on antimuscarinic therapy—males vs females OR 3.24 (95% CI 1.18–8.92, *P* = 0.02), but adjusted analysis indicated the effect was partially explained by age, SCI level, and AIS Grade—males vs females aOR 2.64 (95% CI 0.80–8.65, *P* = 0.11; Table 3).

Sensitivity analyses using a complete case approach produced results similar to those of the multiple imputation analyses for the Pdet_max_ storage ≥40 cmH_2_O, UI symptoms, antimuscarinic use, and DOI outcomes (Tables S4 and S5).

## Discussion

In our cohort, only 9% of females reached a Pdet_max_ storage ≥40 cmH_2_O within the first year after SCI, compared to 55% of males. Overall, the Pdet_max_ storage values were consistently lower in females. One possible explanation for this disparity is the notably lower DOLPP in females, which may be attributed to anatomical differences, particularly the shorter urethra that results in reduced resistance to urinary flow. In contrast, in males the prostate can provide additional resistance [18].

In our cohort, there was no statistically significant difference in reported UI between females and males, likely due to these anatomical/physiological differences between the sexes. Consequently, the cumulative incidence of antimuscarinic therapy was similar between the sexes, suggesting that management decisions are influenced to a considerable extent by individual symptoms as opposed to purely relying on storage pressure values.

A recent publication on the same cohort demonstrated that the risk of initial occurrence of a Pdet_max_ storage ≥40 cmH_2_O within the first year after SCI was highest during the first 3 months after SCI, suggesting that baseline UDI should be performed within this critical period to enable early diagnosis and treatment of NLUTD. Moreover, male sex was identified as a risk factor for developing unfavourable UDI parameters [10]. In light of these results and our findings, we advocate for intensified research efforts to develop sex‐tailored UDI follow‐up schedules and NLUTD management.

In recent years, awareness of the impact of sex‐ and gender‐related differences has grown, influencing both research and therapeutic management strategies [19]. Historically, the SCI population has been predominantly male with a male‐to‐female ratio exceeding 4:1 [20]. Consequently, SCI study populations are mainly comprised of males [21]. However, over the decades the incidence rate among females has increased, largely due to a rise in injuries among the elderly, where cases are more evenly distributed between the sexes [22]. Recent epidemiological data estimate that the incidence of SCI incidence is now approaching a more balanced distribution between females and males [23]. Research on functional and neurological recovery is a subset of SCI research that has been evaluating sex differences for two decades [24, 25], while sex differences in urological care after SCI have been identified as a significant knowledge gap [26]. A few studies examining bladder emptying methods after SCI have shown that clean intermittent self‐catheterisation is more frequently used in males than in females, while the rate of indwelling catheter use is higher among females [27, 28]. The findings regarding suprapubic catheter were not supported by the present data set, possibly due to the fact that females in our cohort predominantly had less severe SCI and a relatively high prevalence of spontaneous voiding. Furthermore, it has been shown females with SCI had worse LUTS, especially UI, and lower bladder‐related satisfaction [27, 29]. The notably lower DOLPP found in this study could provide a potential explanation for the underlying cause of the higher rates of UI in female individuals with SCI.

### Strengths and Limitations

To the best of our knowledge, this is the first study to assess sex differences in urodynamic outcomes using a standardised UDI schedule. Strengths of the study include the utilisation of a population‐based recruitment strategy and the high participation rate; however, the study also has several limitations. There were few females with SCI, especially in younger age groups. Also, compared to males, there were fewer females with more severe (AIS Grade A–C) injury, likely due to the aforementioned sex‐specific trends in SCI incidence, and the female group had a lower (although not statistically significant) percentage of cervical SCI than the male group. Therefore, the study was not adequately powered to fully investigate interaction terms, the potential mediating or moderating roles of age and SCI characteristics, or sex‐specific risk factors for the outcomes of interest. Although causal inference strategies were applied, these analyses should be seen as a first step, and further research in large population‐based data sets is needed to confirm the conclusions and evaluate the generalisability of the results to other settings and to groups that are not included in the present sampling frame (e.g., patients with polyneuropathy). Moreover, the effect of pre‐existing UI could only be investigated in females. This study used the established cut‐off of Pdet_max_ storage ≥40 cmH_2_O as a risk threshold for UUT deterioration after SCI [7]. However, evidence supporting this cut‐off value in the SCI population is limited, suggesting that this threshold could potentially be set too low or too high [8, 9]. In addition, data on renal function were collected by indication, with a relatively large amount of missing data at later time points, further studies with standardised data collection regarding renal insufficiency are needed to provide definitive evidence on renal outcomes in this population. Furthermore, the medical history data did not differentiate between SUI and urgency UI. In addition, UI has a multifactorial aetiology, but information on associated risk factors (e.g., obesity, smoking, and obstetric history) was lacking. Similarly, the information regarding concomitant urological and neurological conditions was limited.

## Conclusions

Within the first year after SCI, females rarely reached a Pdet_max_ storage pressure ≥40 cmH_2_O and generally had lower Pdet_max_ storage and DOLPP than males. Despite these differences in bladder pressure, the prevalence of UI was found to be similar between the sexes. These findings highlight the need for an increased emphasis on research to develop strategies for sex‐tailored management of LUT dysfunction in patients with SCI to address the unique physiological differences and ensure effective treatment for both sexes.

## Funding

Financial support was provided by the Swiss National Science Foundation (SNSF, 179644). The funding body did not play a role in the design of the study, the analysis and interpretation of data or in writing the manuscript.

## Disclosure of Interests

The authors have no direct or indirect commercial financial incentive associated with publishing the article.

## Author Contributions

Study conceptualisation and design: Veronika Birkhäuser, Collene E. Anderson, Marko Kozomara, Martina D. Liechti, Thomas M. Kessler. Data collection: Veronika Birkhäuser, Oliver Gross, Lorenz Leitner, Ulrich Mehnert, Lara Stächele. Analysis of UDI: Veronika Birkhäuser, Marko Kozomara. Statistical analysis: Collene E. Anderson, Martin W.G. Brinkhof. Interpretation of the data: Veronika Birkhäuser, Collene E. Anderson, Marko Kozomara, Martin W.G. Brinkhof, Oliver Gross, Lorenz Leitner, Martina D. Liechti, Ulrich Mehnert, Lara Stächele, Thomas M. Kessler. Drafting the manuscript: Veronika Birkhäuser, Collene E. Anderson, Thomas M. Kessler. Critical revision of the manuscript: Veronika Birkhäuser, Collene E. Anderson, Marko Kozomara, Martin W.G. Brinkhof, Oliver Gross, Lorenz Leitner, Martina D. Liechti, Ulrich Mehnert, Lara Stächele, Thomas M. Kessler.

## Supporting information


**Fig. S1.** Causal diagram depicting the relationship between sex and Pdet_max_ during the storage phase.
**Table S1.** UDI findings, stratified by sex.
**Table S2.** Factors associated with Pdet_max_ during the storage phase, stratified according to antimuscarinic use at the time of UDI.
**Table S3.** Factors associated with DOLPP stratified according to antimuscarinic use at the time of UDI.
**Table S4.** Factors associated with DOI during the first year after SCI.
**Table S5.** Factors associated with Pdet_max_ ≥40 cmH_2_O during the storage phase, UI symptoms, and antimuscarinics during the first year after SCI (complete case analysis).
