# Supplementary material for: Male sex is a risk factor for detrusor pressure jeopardising the upper urinary tract in patients with spinal cord injury
Source: BJU Int. 2025 Sep 18;136(6):1090–7. doi: 10.1111/bju.16925 (PMC12606518; doi:10.1111/bju.16925)
Supplement: Supplementary file 1 — Fig. S1. Causal diagram depicting the relationship between sex and Pdetmax during the storage phase. Table S1. UDI findings, stratified by sex. Table S2. Factors associated with Pdetmax during the storage phase, stratified according to antimuscarinic use at the time of UDI. Table S3. Factors associated with DOLPP stratified according to antimuscarinic use at the time of UDI. Table S4. Factors associated with DOI during the first year after SCI. Table S5. Factors associated with Pdetmax ≥40 cmH2O during the storage phase, UI symptoms, and antimuscarinics during the first year after SCI (complete case analysis). [file BJU-136-1090-s001.pdf]

Supplementary Material

V. Birkhäuser, C.E. Anderson, M. Kozomara, M.W.G. Brinkhof, O. Gross, L. Leitner, M.D. Liechti, U. Mehnert, L. Stächele and T.M. Kessler. "Male sex is a risk factor for detrusor pressure jeopardising the upper urinary tract in patients with spinal cord injury." ***BJUI*****, 2025.**

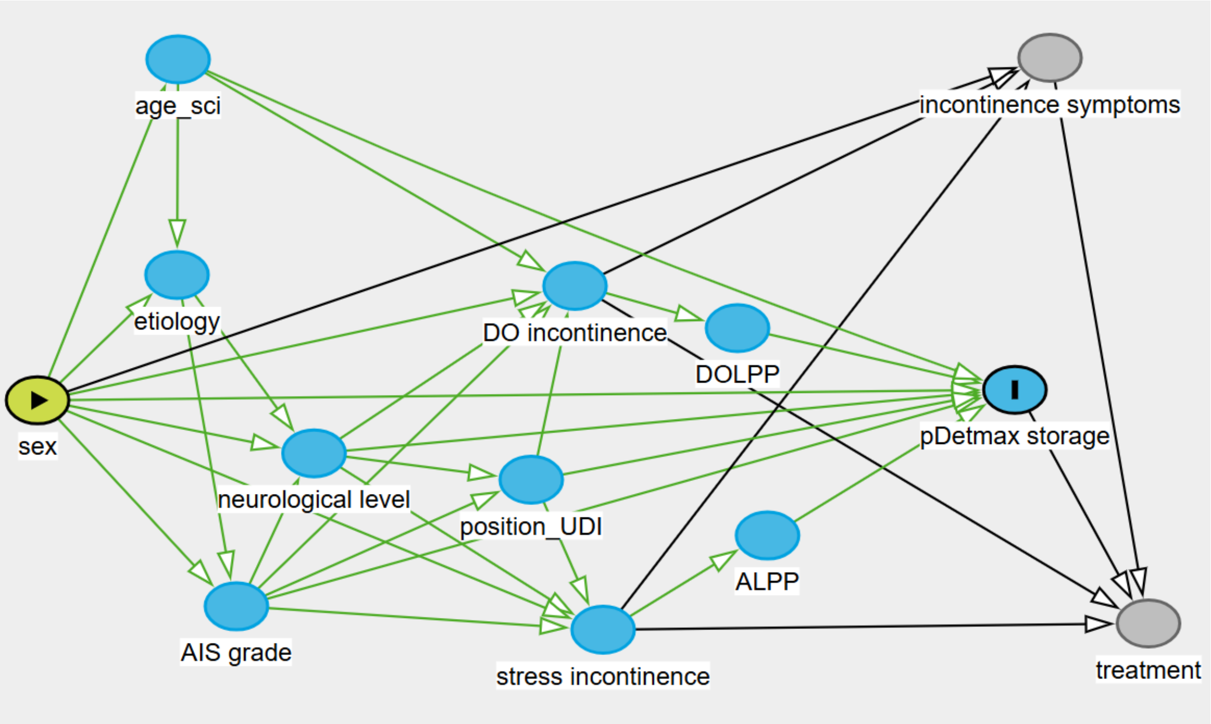

Figure S1. Causal diagram depicting the relationship between sex and maximum detrusor pressure during the storage phase. The directed acyclic graphic [DAG] used to inform model building strategy, depicting the hypothesised relationships between sex (exposure) and maximum detrusor pressure during the storage phase (pDetmax storage, outcome), as well other key factors involved in the causal process. The diagram was developed using DAGitty software.[1] Arrows are only inserted when a causal relationship is hypothesised and always go from cause to effect. Etiology was not included in the final analysis as its effects on the outcome of interest are likely to be mainly indirect over SCI characteristics and the small sample size and necessitated a parsimonious model. Due to similar sample size concerns, the model is broken down into multiple less complex analyses of the component pieces. Furthermore, analyses of detrusor pressure were stratified on antimuscarinic use status in order to avoid collider bias.

ALPP=abdominal leak point pressure DO=detrusor overactivity, DOLPP=detrusor overactivity leak point pressure, pDetmax=maximum detrusor pressure, UDI=urodynamic investigation

[1]Textor, J., van der Zander, B., Gilthorpe, M. S. et al.: Robust causal inference using directed acyclic graphs: the R package 'dagitty'. Int J Epidemiol, 45: 1887, 2016.

Table S1. Urodynamic investigation findings, stratified by sex.

Parameter	Female 1-month follow-up	Male 1-month follow-up	Female 3-month follow-up	Male 3-month follow-up	Female 6-month follow-up	Male 6-month follow-up	Female 12-month follow-up	Male 12-month follow-up
**Urodynamics Performed**	**31 (94)**	**59 (92)**	**30 (91)**	**55 (86)**	**26 (79)**	**49 (77)**	**23 (70)**	**50 (78)**
Time from SCI to UDI (Days), median (Q1-Q3)	30 (24-36)	30 (24-37)	82.5 (78-89)	86 (80-91)	173.5 (169-177)	175 (170-177)	364 (357-381)	365.5 (355-377)
Sitting position during UDI, n (%)	24 (77)	33 (56)	23 (77)	38 (69)	23 (88)	37 (76)	21 (91)	37 (74)
**Filling Cystometry**								
Sensation present, n (%)	28 (90)	48 (81)	29 (97)	44 (80)	22 (85)	39 (80)	19 (83)	39 (78)
First sensation of filling volume (mL), median (Q1-Q3)	160 (133-273)	255 (123-365)	323 (200-383)	293 (173-401)	216 (170-393)	259 (165-315)	268 (185-360)	283 (170-410)
First desire to void volume (mL), median (Q1-Q3)	320 (205-358)	360 (253-510)	333 (250-430)	420 (278-510)	326 (229-434)	363 (285-450)	388 (260-515)	455 (225-525)
Strong desire to void volume (mL), median (Q1-Q3)	435 (373-558)	516 (376-664)	445 (365-635)	525 (335-700)	392 (330-658)	528 (343-688)	496 (386-750)	521 (320-675)
Max Cystometric Capacity (mL), median (Q1-Q3)	548 (405-625)	640 (460-863)	530 (415-710)	685 (460-935)	528 (413-693)	565 (423-830)	588 (370-840)	668 (435-825)
Compliance (mL/cmH_2O), median (Q1-Q3)	98 (49-243)	65 (40-125)	105 (46-255)	86 (44-171)	80 (41-206)	64 (41-122)	105 (47-360)	69 (46-100)
DO present, n (%)	19 (61)	45 (76)	15 (50)	48 (87)	15 (58)	46 (94)	15 (65)	42 (84)
DO start volume (mL), median (Q1-Q3)	210 (155-490)	260 (205-385)	205 (200-375)	363 (200-540)	305 (135-415)	373 (185-470)	395 (185-460)	380 (185-495)
DO incontinence present, n (%)	8 (26)	24 (41)	8 (27)	15 (27)	8 (31)	21 (43)	4 (17)	12 (24)
DO leak point pressure (cmH_2O), median (Q1-Q3)	17 (13-19)	35 (25-69)	21 (12-22)	33 (17-49)	14 (13-15)	33 (19-46)	11 (4-12)	27 (13-37)
Volume leaked (DOI) (mL), median (Q1-Q3)	85 (20-400)	50 (20-89)	75 (38-229)	40 (33-60)	75 (43-98)	24 (10-48)	108 (25-303)	45 (13-56)
Stress urinary incontinence present, n (%)	4 (13)	2 (3)	4 (13)	1 (2)	2 (8)	3 (6)	3 (13)	3 (6)
Abdominal leak point pressure (cmH_2O), median (Q1-Q3)	23 (2-81)	70 (70-70)	5 (1-10)	0 (0-0)	19 (1-33)	32 (7-57)	12 (11-16)	10 (6-59)
Volume leaked (SUI), median (Q1-Q3)	45 (20-48)	5 (5-5)	20 (13-60)	15 (15-15)	40 (5-45)	28 (20-70)	29 (15-45)	21 (9-30)
Max detrusor pressure - storage (cmH_2O), median (Q1-Q3)	9 (4-13)	28 (9-42)	10 (4-17)	28 (14-45)	12 (4-21)	31 (16-39)	15 (4-18)	25 (15-37)
Max detrusor pressure - storage ≥40 cmH_2O, n (%)	1 (3)	20 (34)	1 (3)	17 (31)	2 (8)	22 (45)	0 (0)	11 (22)
**Pressure Flow**								
Detrusor sphincter dyssynergia, n (%)	29 (94)	40 (68)	30 (100)	45 (82)	25 (96)	38 (78)	20 (87)	40 (80)
Spontaneous voiding, n (%)	16 (52)	20 (34)	18 (60)	28 (51)	19 (73)	22 (45)	16 (70)	22 (44)
Maximum flow rate (mL/sec), median (Q1-Q3)	28 (15-40)	13 (8-17)	16 (10-36)	13 (10-17)	15 (11-28)	15 (8-20)	21 (13-32)	11 (8-14)
Detrusor pressure during maximum flow rate, median (Q1-Q3)	34 (18-46)	52 (31-68)	25 (16-37)	36 (29-52)	25 (11-40)	37 (22-52)	26 (21-31)	40 (32-54)
Max detrusor pressure - voiding (cmH_20), median (Q1-Q3)	36 (19-75)	53 (41-102)	27 (18-54)	48 (34-76)	32 (17-51)	45 (32-61)	36 (31-47)	50 (37-66)
Voided volume (mL), median (Q1-Q3)	343 (105-625)	240 (120-518)	503 (110-730)	218 (100-425)	300 (220-500)	280 (160-530)	425 (215-688)	253 (110-510)
Post-void residual (mL), median (Q1-Q3)	195 (0-455)	200 (45-375)	180 (0-330)	260 (65-490)	225 (50-400)	190 (100-310)	110 (20-323)	235 (140-340)
Voiding profile								
Spontaneous voiders (excluding abdominal straining), n (%)	13 (42)	20 (34)	13 (43)	27 (49)	13 (50)	21 (43)	7 (30)	19 (38)
Non-obstructive	9 (69)	7 (35)	9 (69)	15 (56)	8 (62)	16 (76)	7 (100)	11 (58)
Equivocal	NA	4 (20)	NA	2 (7)	NA	1 (5)	NA	5 (26)
Obstructive	0 (0)	5 (25)	0 (0)	4 (15)	5 (38)	3 (14)	0 (0)	3 (16)
Autonomic dysreflexia present, n (%)	8 (26)	30 (51)	8 (27)	32 (58)	11 (42)	28 (57)	10 (43)	31 (62)

Abbreviations: DO=detrusor overactivity, DOI=detrusor overactivity incontinence, Q=quartile, SCI=spinal cord injury, SUI=stress urinary incontinence, UDI=urodynamic investigation

Table S2. Factors associated with maximum detrusor pressure (pDetmax) during the storage phase, stratified according to antimuscarinic use at the time of urodynamics. Results from uni- and multivariable linear mixed-effects regression models, with a natural log transformation of the outcome variable to account for a right-skewed distribution. Storage pDetmax no antimuscarinic treatment group: n=97 patients, with 439 urodynamic fillings; storage pDetmax antimuscarinic treatment group: n=50 patients and 174 urodynamic fillings.

**Determinant**	**pDetmax Storage, no antimuscarinics, coefficient (95% CI)**	***p***	**pDetmax Storage, antimuscarinics, coefficient (95% CI)**	***p***	**pDetmax Storage, no antimuscarinics, adjusted coefficient (95% CI)**	***p***	**pDetmax Storage, antimuscarinics, adjusted coefficient (95% CI)**	***p***
**Sex**		<0.0001		0.013		<0.0001		<0.001
Female	REF		REF		REF		REF	
Male	0.99 (0.64, 1.35)		0.76 (0.16, 1.36)		0.78 (0.44, 1.12)		1.04 (0.44, 1.63)	
**Age at SCI [years]**	0.00 (-0.01, 0.02)	0.52	0.00 (-0.02, 0.02)	0.99	0.01 (0.00, 0.02)	0.29	0.01 (-0.01, 0.03)	0.58
**Neurological Level**		0.041		0.031		0.021		0.53
Cervical	0.67 (0.15, 1.20)		1.09 (0.23, 1.95)		0.47 (0.14, 0.81)		-0.04 (-0.74, 0.65)	
Thoracic	0.35 (-0.16, 0.86)		1.12 (0.25, 1.99)		0.23 (-0.08, 0.54)		0.19 (-0.39, 0.78)	
Lumbar	REF		REF		REF		REF	
**AIS Grade**		0.24		0.97		0.10		0.97
A	0.39 (-0.08, 0.86)		0.07 (-0.58, 0.72)		0.30 (-0.12, 0.72)		0.06 (-0.49, 0.60)	
B / C	0.25 (-0.24, 0.73)		-0.03 (-0.67, 0.62)		0.28 (0.01, 0.56)		0.04 (-0.53, 0.62)	
D	REF		REF		REF		REF	
**Detrusor overactivity incontinence**		<0.0001		<0.01		<0.0001		0.013
Yes	0.81 (0.59, 1.02)		0.55 (0.19, 0.91)		0.71 (0.49, 0.93)		0.48 (0.10, 0.87)	
No	REF		REF		REF		REF	
**Stress urinary incontinence**		<0.0001		<0.0001		<0.001		0.052
Yes	-0.87 (-1.26, -0.48)		-0.79 (-1.18, -0.41)		-0.57 (-0.89, -0.24)		-0.62 (-1.24, 0.01)	
No	REF		REF		REF		REF	
**Time to CMG [months]**	0.00 (-0.02, 0.03)	0.72	-0.02 (-0.04, 0.00)	0.042	0.01 (-0.01, 0.04)	0.30	-0.02 (-0.04, 0.00)	0.07
**Filling**		0.56		0.16		0.43		0.18
Filling 1	REF		REF		REF		REF	
Filling 2	-0.03 (-0.12, 0.07)		-0.11 (-0.27, 0.04)		-0.03 (-0.12, 0.05)		-0.10 (-0.24, 0.04)	
**Position during UDI**		0.12		0.87		0.78		0.31
Sitting	REF		REF		REF		REF	
Lying down	0.25 (-0.06, 0.56)		-0.03 (-0.45, 0.38)		0.04 (-0.25, 0.33)		-0.20 (-0.58, 0.19)	

AIS=American Spinal Cord Injury Association Impairment Scale, CI=confidence interval; CMG=cystometrogram, pDetmax=maximum detrusor pressure, SCI=spinal cord injury, UDI=urodynamic investigation

Table S3. Factors associated with detrusor overactivity leak point pressure (DOLPP), stratified according to antimuscarinic use at the time of urodynamics. Results from uni- and multivariable linear mixed-effects regression models, with a natural log transformation of the outcome variable to account for a right-skewed distribution. DOLPP no antimuscarinic treatment group: n=29 patients with 60 urodynamic fillings, DOLPP antimuscarinic treatment group: n=16 patients with 39 urodynamic fillings. Coefficients for neurological level and AIS grade should be interpreted with care, as the analysis data set does not include any patients with cervical AIS grade A,B,C SCI due to measurement in lying position.

**Determinant**	**DOLPP, no antimuscarinics, coefficient (95% CI)**	***p***	**DOLPP, antimuscarinics, coefficient (95% CI)**	***p***	**DOLPP, no antimuscarinics, adjusted coefficient (95% CI)**	***p***	**DOLPP, antimuscarinics, adjusted coefficient (95% CI)**	***p***
**Sex**		0.014		<0.0001		<0.01		<0.0001
Female	REF		REF		REF		REF	
Male	0.52 (0.11, 0.93)		1.15 (0.67, 1.63)		0.79 (0.32, 1.27)		1.35 (0.88, 1.81)	
**Age at SCI [years]**	0.01 (-0.01, 0.03)	0.23	-0.01 (-0.03, 0.00)	0.15	0.02 (0.00, 0.03)	0.054	0.01 (0.00, 0.03)	0.15
**Neurological Level**		0.28		0.30		0.99		0.98
Cervical	0.16 (-0.69, 1.00)		0.86 (-0.76, 2.47)		0.05 (-0.64, 0.73)		-0.10 (-1.17, 0.96)	
Thoracic	-0.26 (-1.11, 0.59)		0.43 (-1.17, 2.02)		0.04 (-0.63, 0.70)		-0.09 (-1.04, 0.87)	
Lumbar	REF		REF		REF		REF	
**AIS Grade**		0.10		0.049		0.12		0.051
A	-0.53 (-1.03, -0.03)		0.18 (-0.30, 0.66)		-0.88 (-1.73, -0.03)		0.30 (-0.33, 0.93)	
B / C	-0.06 (-0.72, 0.60)		-0.63 (-1.43, 0.17)		-0.22 (-0.89, 0.46)		-0.56 (-1.06, -0.07)	
D	REF		REF		REF		REF	
**Time to CMG [months]**	0.01 (-0.03, 0.05)	0.53	-0.03 (-0.05, 0.00)	0.039	0.01 (-0.02, 0.05)	0.43	-0.03 (-0.05, -0.01)	<0.001
**Filling**		0.37		0.35		0.46		0.38
Filling 1	REF		REF		REF		REF	
Filling 2	0.06 (-0.08, 0.20)		-0.08 (-0.25, 0.09)		0.05 (-0.09, 0.19)		-0.07 (-0.23, 0.09)	

AIS=American Spinal Cord Injury Association Impairment Scale; CI=confidence interval; CMG=cystometrogram; DOLPP=detrusor overactivity leak point pressure, SCI=spinal cord injury

Table S4. Factors associated with detrusor overactivity (DO) incontinence during the first year after SCI. Results from uni- and multivariable logistic regression analyses with presence of the respective outcome coded as '1'. The analysis was run once imputing missing data with multiple imputation using chained equations (MI), and once as complete case (CC). Age at injury, neurological level and AIS grade are all potential mediators for the effect of sex on the outcomes.

**Determinant**	**DO Incontinence During the First Year after SCI (MI), OR (95% CI)**	***p***	**DO Incontinence During the First Year after SCI (MI), aOR (95% CI)**	***p***	**DO Incontinence During the First Year after SCI (CC), OR (95% CI)**	***p***	**DO Incontinence During the First Year after SCI (CC), aOR (95% CI)**	***p***
**Sex**		0.26		0.22		0.19		0.10
Female	REF		REF		REF		REF	
Male	1.68 (0.68 - 4.15)		1.97 (0.67 - 5.81)		1.85 (0.74 - 4.62)		2.43 (0.84 - 7.04)	
**Age at SCI**	1.03 (1.00 - 1.06)	0.029	1.04 (1.01 - 1.07)	0.016	1.03 (1.01 - 1.06)	0.019	1.04 (1.01 - 1.07)	<0.01
**Neurological Level**		0.33		0.39		0.13		0.11
Cervical	2.30 (0.64 - 8.20)		2.76 (0.51 - 14.90)		2.74 (0.74 - 10.11)		3.32 (0.65 - 16.81)	
Thoracic	2.66 (0.70 - 10.14)		3.37 (0.59 - 19.20)		4.00 (1.04 - 15.34)		5.63 (1.12 - 28.38)	
Lumbar	REF		REF		REF		REF	
**AIS Grade**		0.14		0.35		0.15		0.37
A	3.59 (1.01 - 12.78)		2.76 (0.65 - 11.78)		3.51 (1.00 - 12.31)		2.53 (0.63 - 10.15)	
B / C	1.35 (0.45 - 4.03)		1.86 (0.41 - 8.46)		1.29 (0.45 - 3.64)		1.77 (0.45 - 7.03)	
D	REF		REF		REF		REF	

AIS=American Spinal Cord Injury Association Impairment Scale; aOR=adjusted odds ratio; CI=confidence interval; DO=detrusor overactivity, OR=odds ratio; SCI=spinal cord injury

Table S5. Factors associated with detrusor pressure (pDetmax) ≥40 cmH_2O during the storage phase, incontinence symptoms, and antimuscarinics during the first year after SCI (complete case analysis). Results from uni- and multivariable logistic regression analyses with presence of the respective outcome coded as '1'. Age at injury, neurological level and AIS grade are all potential mediators for the effect of sex on the respective outcomes.

	**pDetmax ≥40 cmH_2O, OR (95% CI)**	***p***	**pDetmax ≥40 cmH_2O, aOR (95% CI)**	***p***	**Incontinence Symptoms, OR (95% CI)**	***p***	**Incontinence Symptoms, aOR (95% CI)**	***p***	**Antimuscarinics, OR (95% CI)**	***p***	**Antimuscarinics, aOR (95% CI)**	***p***
**Sex**		<0.001		<0.001		0.32		0.58		0.022		0.17
Female	REF		REF		REF		REF		REF		REF	
Male	17.38 (3.99 - 75.60)		16.63 (4.02 - 68.75)		0.62 (0.25 - 1.58)		0.74 (0.24 - 2.21)		3.33 (1.19 - 9.33)		2.40 (0.69 - 8.31)	
**Age at SCI**	1.02 (0.99 - 1.06)	0.23	1.01 (0.98 - 1.05)	0.52	1.02 (0.99 - 1.04)	0.24	1.02 (0.99 - 1.05)	0.22	1.02 (0.99 - 1.05)	0.21	1.02 (0.99 - 1.06)	0.16
**Neurological Level***		0.10		0.22		0.026		0.08		0.87		0.92
Cervical	3.87 (0.85 - 17.63)		4.31 (0.82 - 22.67)		0.73 (0.21 - 2.47)		0.41 (0.11 - 1.61)		1.43 (0.35 - 5.83)		1.51 (0.22 - 10.51)	
Thoracic	1.12 (0.28 - 4.43)		4.01 (0.63 - 25.43)		2.92 (0.81 - 10.52)		1.39 (0.34 - 5.74)		1.39 (0.33 - 5.80)		1.39 (0.21 - 9.14)	
Lumbar	REF		REF		REF		REF		REF		REF	
**AIS Grade***		0.28		0.17		0.040		0.09		0.026		0.07
A	0.53 (0.13 - 2.17)		0.46 (0.12 - 1.72)		2.71 (0.82 - 8.96)		2.59 (0.64 - 10.58)		12.80 (1.54 - 106.29)		9.66 (1.15 - 81.25)	
B / C	1.60 (0.38 - 6.79)		2.62 (0.52 - 13.22)		0.48 (0.17 - 1.40)		0.42 (0.13 - 1.43)		2.88 (0.82 - 10.15)		2.77 (0.63 - 12.16)	
D	REF		REF		REF		REF		REF		REF	

* Neurological level and AIS grade reflect neurological status within 40 days of SCI. In 5 cases with missing data at the 1-month time point, information was taken from the next time point with available data.
AIS=American Spinal Cord Injury Association Impairment Scale; aOR=adjusted odds ratio; CI=confidence interval; OR=odds ratio; pDetmax=maximum detrusor pressure; SCI=spinal cord injury
